# SB202190 Predicts BRAF-Activating Mutations in Primary Colorectal Cancer Organoids via Erk1-2 Modulation

**DOI:** 10.3390/cells12040664

**Published:** 2023-02-20

**Authors:** Delfina Costa, Roberta Venè, Simona Coco, Luca Longo, Francesca Tosetti, Stefano Scabini, Luca Mastracci, Federica Grillo, Alessandro Poggi, Roberto Benelli

**Affiliations:** 1 SSD Oncologia Molecolare e Angiogenesi, IRCCS Ospedale Policlinico San Martino, Largo Rosanna Benzi 10, 16132 Genova, Italy; 2 UO Tumori Polmonari, IRCCS Ospedale Policlinico San Martino, Largo Rosanna Benzi 10, 16132 Genova, Italy; 3 UO Chirurgia Generale ad Indirizzo Oncologico, IRCCS Ospedale Policlinico San Martino, Largo Rosanna Benzi 10, 16132 Genova, Italy; 4 UO Anatomia Patologica, IRCCS Ospedale Policlinico San Martino, Largo Rosanna Benzi 10, 16132 Genova, Italy; 5 Dipartimento di Scienze Chirurgiche e Diagnostiche Integrate (DISC), Anatomia Patologica, Università di Genova, 16132 Genova, Italy

**Keywords:** colorectal cancer, organoid, SB202190, BRAF, Erk1-2

## Abstract

The p38 inhibitor SB202190 is a necessary component of the medium used for normal colorectal mucosa cultures. Sato et al. suggested that the primary activity of SB202190 may be EGFR signaling stabilization, causing an increased phosphorylation of Erk1-2 sustaining organoid proliferation. However, the growth of some colorectal cancer (CRC)-derived organoid cultures is inhibited by this molecule via an unknown mechanism. We biochemically investigated SB202190 activity on a collection of 25 primary human CRC organoids, evaluating EGFR, Akt and Erk1-2 activation using Western blot. We found that Erk1-2 phosphorylation was induced by SB202190 in 20 organoid cultures and inhibited in 5 organoid cultures. A next-generation sequencing (NGS) analysis revealed that the inhibition of p-Erk1-2 signaling corresponded to the cultures with BRAF mutations (with four different hits, one being undescribed), while p-Erk1-2 induction was apparently unrelated to other mutations involving the EGFR pathway (Her2, KRAS and NRAS). We found that SB202190 mirrored the biochemical activity of the BRAF inhibitor Dabrafenib, known to induce the paradoxical activation of p-Erk1-2 signaling in BRAF wild-type cells. SB202190 was a more effective inhibitor of BRAF-mutated organoid growth in the long term than the specific BRAF inhibitors Dabrafenib and PLX8394. Overall, SB202190 can predict BRAF-activating mutations in patient-derived organoids, as well as allowing for the identification of new BRAF variants, preceding and enforcing NGS data.

## 1. Introduction

The EGFR pathway is a major target for driving mutations and specific drug design in many tumors. EGFR is able to activate several intracellular signaling pathways, affecting cell proliferation, metabolism, differentiation and stress resistance. The main pathways mediating these effects are the GRB2-SOS-RAS-RAF-Mek-Erk pathway, directly triggered by EGFR, and the PI3K-PDK1-Akt-mTOR-S6K pathway, mediated by EGFR-HER2 heterodimers [1]. In colorectal cancer (CRC), KRAS mutations are prevalent and affect the prognosis of patients with metastatic disease, conferring resistance to Cetuximab anti EGFR therapy. Mutated KRAS allows for the EGFR-independent activation of the downstream BRAF/CRAF-Mek-Erk pathway [2]. BRAF mutations are less frequent (11% of sporadic CRC) and mainly associated with right-sided, microsatellite-unstable tumors with mucinous histology [3]. BRAF V600 point mutations (V600E being the most frequent) account for 70–78% of the total BRAF mutations in CRC and create active monomers not needing homo- or hetero-dimerization with CRAF to activate the Mek-Erk pathway [4]. This constitutive activation exerts a strong pathogenic effect on patient overall survival, especially when associated with a microsatellite-stable phenotype [5]. Many other BRAF-activating mutations have been described, and these mutants usually create stable dimers that can show amplified (type II) or reduced (type III) kinase activity, with the latter relying on the upstream activation of EGFR or KRAS mutations to exert a pathogenic effect [4]. These BRAF mutants are associated with left-sided CRC in young patients, usually linked to a more favorable outcome [4]. Due to the unique pathogenic role of BRAF V600E, other BRAF mutations are less investigated in patients with CRC, even though they could be true therapeutic targets. Moreover, the Akt pathway plays a direct role in CRC progression. Frequent mutations can affect PIK3CA and/or PTEN, with the former acting as a signaling agonist and the latter being depleted due to its inhibitory activity on this pathway. While both mutations show a negative prognostic impact, they do not apparently predict Cetuximab resistance in mCRC [6].

Patient-derived organoids represent an outperforming model for theranostic approaches to CRC [7]. These 3D micro-tumor cultures recapitulate the original primary tumor and can be used for specific diagnostic and personalized patient therapy testing [8,9]. The complex cocktail of molecules used to culture CRC organoids does not have a completely defined formula, and some additives have been selected on a “it works” basis, without the full knowledge of their biochemical activity [10]. Indeed, one controversial component of the CRC organoid medium is SB202190, a small-molecule, pyridinyl imidazole p38 MAP kinase inhibitor that directly binds p38 MAP kinases in the ATP binding pocket. Its addition to the organoid culture medium is able to empower Erk1-2 signaling in normal colon mucosa cultures [10], but it shows unpredictable, opposite effects on CRC organoids [11].

In the first Sato and Clevers co-authored paper [10], SB202190 was used at the 10 µM concentration for a normal colon mucosa culture. When the culture cocktail was eventually tested on CRC organoids, Sato’s group continued to use SB202190 at the 10 µM concentration, noticing that some cultures were inhibited, while other ones were unaffected or needed its presence [11]. In a parallel study published by Clevers’ group, SB202190 toxicity was not reported, but the drug was lowered to the 3 µM concentration in all cultures [12]. Sato and colleagues noticed that the need for exogenous EGF and SB202190 co-occurred in some organoids and that SB202190 was able to stabilize EGFR phosphorylation, elevating Erk1-2 signaling [11]. However, the involvement of p38 inhibition as a trigger of this phenomenon was not proven. According to these observations, SB202190 was proposed to be an Erk1-2 activator that could be useful for organoid cultures not showing KRAS- or BRAF-activating mutations, thus relying on EGFR activity. No explanation was given for its inhibitory effect, though CRC inhibition would be quite a valuable endpoint.

Herein, we systematically assessed, on a series of CRC organoids, the influence of SB202190 on the EGFR signaling pathway. We found that this drug can identify unique BRAF-activating mutations, eventually validated using NGS.

## 2. Materials and Methods

Patient recruitment: 70 subjects were recruited at the unit of Oncologic Surgery and Implantable Systems after giving their written informed consent (EC approvals by the Ethics Committee of San Martino Hospital no. 4/2011 and Regional Ethics Committee Regione Liguria PR163REG2014, renewed in 2017). Tumors, staged as I (8), II (29), III (25) and IV (8) according to the UICC TNM classification, were located in the ascending (R = 33), transverse (T = 11), descending (L = 3) and sigmoid (S = 6) colon, and rectum (RT = 17) (Table S1).

Tissue processing for primary organoid cultures: An expert pathologist collected a representative fragment of the invasive tumor from the surgical specimen. Organoids were established as already described [13]. Briefly, the sample tissue was minced and enzymatically digested at 37 °C in Leibovitz’ L15 medium (Gibco), containing EGTA 0.5 mM, Penicillin 100 UI/mL and Streptomycin 100 ug/mL (EuroClone), Gentamycin 5 ug/mL (Sigma) and Collagenases I and II from Clostridium 1.5–3 mg/mL (Gibco, type I cat 17100-017 and type II cat 17101-015; activity 200 UI/mg). At the end of digestion (45 min), the tissue suspension was washed three times in RPMI (Gibco) 10% FCS (EuroClone, ESC-tested cat ECS0196L) and passed through a 100 µm strainer (Sarstedt, cat 83.3945.100). The filtered tumor fragments were washed two times and included in geltrex (LDEV-free, HESc-qualified cat A1413302), plating 6–7, 5 µ domes per well in a 24 multi-well plate (Euroclone cat ET3024). After 25 min at 37 °C, the polymerized domes were immersed in 500 microliters per well of medium. Medium with or without SB202190 10 µM (p38 inhibitor, Selleckchem cat S1077, purity ≥ 99%) was tested to determine the condition that favors organoid growth for each primary culture, according to Fujii et al. [11].

Culture/experimental conditions: The medium was changed every other day, and the cultures showing active proliferation were split by trypsin, 1:2 to 1:8, in new geltrex domes every 7–10 days. The medium did not contain serum, cell-derived supernatants or recombinant growth factors, with the exception of animal-free EGF, to limit spurious signaling. The organoids were tested within 2–8 culture splits. Drugs tested: SB202190 10 µM, Dabrafenib mesylate 1 µM (BRAFV600E inhibitor, Selleckchem cat S5069, purity > 99%); PLX8394 1 µM (BRAFV600E monomer and dimer inhibitor, Selleckchem cat S7965, purity > 99%); and BIRB796/Doramapimod 0.2 µM (p38 inhibitor, Medchemexpress cat HY-10320, purity 99.88%).

Samples for Western blot were treated in a fresh culture medium, with the drugs, for 2 h. Organoids in 48 h old medium were used as a basal signaling control. Some tests were performed in an EGF-free medium. To eliminate single-organoid variability, each experimental point was formed by a pool of 6–8 geltrex domes, each containing 50–100 organoids (condition used both for biochemistry and NGS).

Immunohistochemistry: CRC organoids were collected by pipetting from the geltrex domes; after fixation in HistoChoice-MB (VWR cat 97060-978) for 24 h, they were pelleted in a 1.5 mL vial (Eppendorf) and included in a drop of agar. The solidified agar drop was treated according to standard procedures for dehydration and paraffin inclusion. Then, 3 µm thick sections were mounted on Superfrost slides (Thermo Scientific), and immunohistochemistry was carried out using an automated Bond RX Immunostainer (Leica). Primary antibody dilutions: anti-Vil1 1:400 (rabbit polyclonal, prestige antibody Sigma cat HPA006885), anti-MUC2 1:400 (mouse mAb, Cell Marque cat MRQ-18) and anti-Chromogranin-A 1:1000 (mouse mAb, Chemicon cat MAB5268).

Western blot: RIPA-buffer-extracted total proteins (8 µg/lane) were resolved on ExpressPlus 8% PAGE gels (GenScript cat M00812) and blotted on PVDF membranes (GE-healthcare). Antibody dilutions: anti-p-EGFR 1:1000 (Tyr1068 D7A5 rabbit mAb, Cell Signaling Technology cat 3777), anti-EGFR 1:1000 (D38B1 rabbit mAb, Cell Signaling Technology cat 4267), anti-p-Akt 1:1000 (Ser473 D9E rabbit mAb, Cell Signaling Technology cat 4060), anti-Akt 1:1000 (rabbit, Cell Signaling Technology cat 9272), anti-p-Erk1-2 1:2000 (Thr202/Tyr204 rabbit, Cell Signaling Technology cat 9101), anti-Erk1-2 1:2000 (rabbit, Cell Signaling Technology cat 9102), anti-rabbit HRP-conjugated secondary antibody 1:4000 (goat, Cell Signaling Technology cat 7074) and anti-beta-actin HRP-conjugated 1:10000 (13E5 rabbit mAb, Cell Signaling Technology cat 5125) used as loading control. Protein bands were detected using a chemiluminescent HRP substrate (Immobilon Western, Millipore) and acquired using Hyperfilm ECL (GE-healthcare).

Western blot lanes were quantified using Image Studio Lite analysis software (LI-COR Biosciences), and phosphorylated proteins were normalized against their specific total protein content and beta-actin; most results are reported as the inhibitor-treated/control ratio of p-EGFR, p-Akt and p-Erk1-2 normalized values.

Mutation analyses using next-generation sequencing (NGS): Genomic DNA (gDNA) was extracted from fresh frozen CRC cells using a QIAamp DNA Mini Kit (Qiagen, Hilden, Germany) and quantified using a Qubit 2.0 Fluorometer (Thermo Fisher Scientific, Carlsbad, CA, USA). The mutational status of 22 known genes (KRAS, EGFR, BRAF, PIK3CA, AKT1, ERBB2, PTEN, NRAS, STK11, MAP2K1, ALK, DDR2, CTNNB1, MET, TP53, SMAD4, FBX7, FGFR3, NOTCH1, ERBB4, FGFR1 and FGFR2) associated with CRC was screened using the Ion PGM platform using the Ion AmpliSeq Colon and Lung Cancer Research Panel v2 (Thermo Fisher Scientific). For each sample, 15 ng of gDNA was amplified according to the protocol for snap-frozen samples [14]. Then, 6 barcoded libraries were multiplexed at a dilution of 100 pM and amplified/enriched using OneTouch™ and the OneTouch™ ES, respectively (Thermo Fisher Scientific). Each template was loaded into a 316v.2 chip and sequenced on Ion PGM™ (Thermo Fisher Scientific). The sequencing data were analyzed using the Ion Torrent Software Suite with the plugin Torrent Variant Caller v5.10.0.18 (Thermo Fisher Scientific) applying somatic, high-stringency parameters. Gene variants were annotated using Ion Reporter™ Software v.5.16.0.2, and the variant effect prediction was assessed using COSMIC [15]. The raw data were loaded into the Sequence Read Archive [16] (BioProject ID: PRJNA759362).

The validation of BRAF gene variants: The BRAF V600E gene variant was confirmed using the QX200 Droplet Digital PCR™ System (Bio-Rad Laboratories, Inc., Hercules, CA, USA) using wet-lab validation mutation assays (FAM-Mutation assay: dHsaCP2000027 and HEX-wild-type assay: dHsaCP2000028). For each reaction, 10 ng of gDNA was amplified in duplicate using the ddPCR Supermix for Probes (No dUTP) following the manufacturer’s instructions. Each PCR run also included a BRAF wild-type sample, a V600E-positive sample and a no-template control. The data were analyzed using QuantaSoft v.1.7.4.0917 software (Bio-Rad Laboratories) in 2D amplitude applying the channel thresholds based on the signals of the no-template, wild-type DNA and V600E-positive controls. The allele fraction for each sample was calculated as the merging of replicates. The BRAF K601E, G469V and InDel (V471dup) variants were validated using Sanger sequencing (Figure S1) as already described [17]. The primers were designed using Primer3 software [18], and their specificities were checked on Primer-BLAST [19]. The primer sequences were BRAF-fwd1: 5′-cttcataatgcttgctctgatagg-3′ and BRAF-rev1: 5′-agcctcaattcttaccatccac-3′ for V600E and K601E; BRAF-fwd2: 5′-acacttggtagacgggactc-3′ and BRAF-rev2: 5′-tgcgaacagtgaatatttcctttg-3′ for G469V and V471 dup.

Proliferation assay: The organoids were trypsinized and split 1:4 to 1:8 in new liquid geltrex, according to the proliferation rate of each primary culture. Single, 5 µL drops of the matrix/cell suspension were plated in new multi-well plates. The organoids were allowed to recover from trypsinization and organize for 48 h. Afterwards, the medium was changed, and the replicates were treated with the drugs at the same concentration used for the Western blots, replacing the medium and treatments every 48 h. Organoid growth was live-monitored using a JuLI^TM^-Stage plate scanner (NanoEntek), and pictures were automatically collected. Organoid size increase was calculated using Image-J to analyze the pictures taken every 12 h. For this purpose, each picture was elaborated by using the *auto threshold* and *analyze particles* plug-ins, and the output parameter *average size* was plotted on Excel files after normalization by the value of the corresponding image taken at time 0 (48 h of culture, just after treatments). At the end of the test, the organoids were marked by C-Live Tox-green (Cytena) and incubated for 4 h. Positivity, corresponding to dead cells, was recorded using JuLI^TM^-Stage and normalized against the total cell population.

Statistical analysis: Control versus treated organoids or wild-type organoids versus BRAF- or KRAS-mutated organoids were analyzed with Student’s *t*-test, using EZR or Excel software.

## 3. Results

### 3.1. Organoid Primary Culture Establishment

The collection involved two cohorts, namely, the experimental and the validation cohorts (Table S1). The first one was composed of 28 samples: 5 cultures could not be established, 14 samples survived in the short-term culture (max 2 passages), and 9 cultures proliferated and could be split and expanded consecutively. The validation cohort (42 cases) included 17 non-established cultures, 7 samples that survived in the short-term culture, and 15 cultures that proliferated and could be expanded. As reported in the Materials and Methods Section, all the primary cultures were established in the presence or absence of SB202190, as Fujii et al. [11] indicated this inhibitor as necessary for the survival of some CRC organoids. In our cohorts, we found only one culture dependent on SB202190 for propagation. On the contrary, we observed an inhibitory effect, also reported by Fujii et al., in several cases. In total, we prospectively obtained 24 continuously proliferating organoid lines from 67 processed CRC samples (35.8% establishment). To the second cohort, we also added three previously established cultures, two of which were chosen for their potential BRAF mutation, based on the pathological reports of the original tumors.

**Figure 1:**
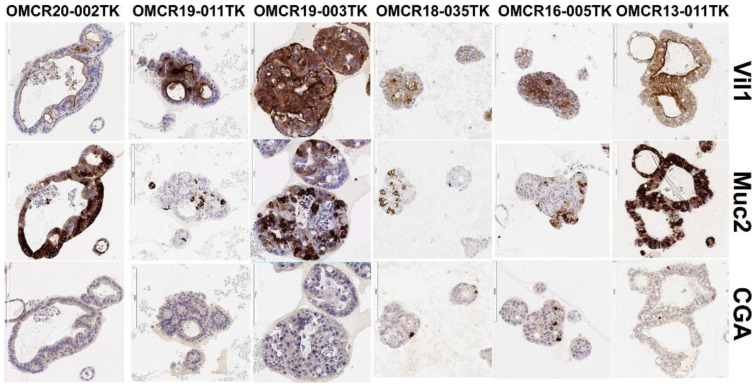
Villin1 (Vil1, enterocyte), Mucin2 (MUC2, goblet cell) and chromogranin-A (CGA, neuroendocrine cell) marker expressions in some representative organoids.

The established continuously proliferating organoid cultures did not show a correlation with a particular stage or grade of the neoplasm of origin. Importantly, the organoids were composed of enterocytes (Vil1+) and goblet (Muc2+) and neuroendocrine (CGA+) cells in different proportions, like true intestinal epithelial crypts, as demonstrated by the IHC analysis (Figure 1). This finding suggests that no in vitro selection of a specific cell lineage occurred and that a multipotent compartment was preserved. Moreover, four out of four organoids derived from CRC with mucinous components showed intense and extended MUC2 staining, suggesting a good fit between the organoids and their originating tumor (not shown).

### 3.2. Influence of SB202190 on the EGFR Signaling Pathway and Its Relation to the Mutational Status of CRC Organoids

Fujii et al. [11] suggested that SB202190’s primary, positive activity may be EGFR signaling stabilization, causing an increased phosphorylation of Erk1-2 sustaining proliferation. We assessed this hypothesis by testing the effects of SB202190 on the phosphorylation of EGFR and the downstream signaling molecules Erk1-2 and Akt to define if this drug could affect EGF signal transduction in our cohort. Our investigation was performed consecutively on the two cohorts. In many samples, p-EGFR was barely detectable by Western blot, as the presence of high-dose EGF in a culture medium can cause a continuous internalization and degradation of the receptor [20]. The basal activation of p-Erk1-2 in the 48 h old medium (-) was very variable, indicating possible autocrine activation loops in some organoid cultures.

**Figure 2:**
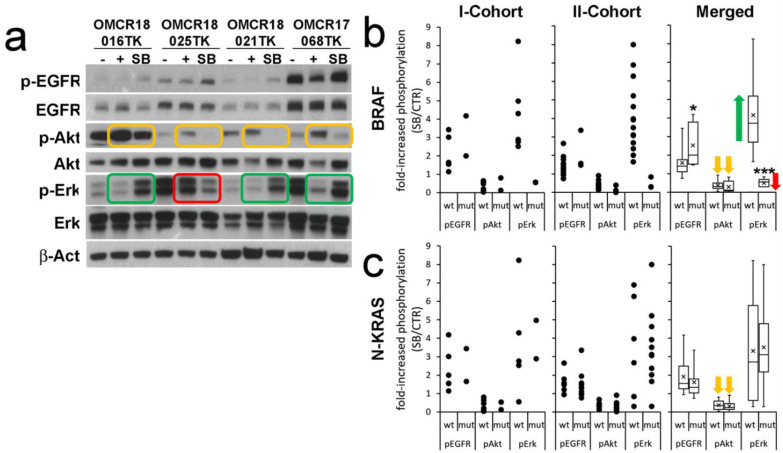
The biochemical signature of SB202190 (SB) on key transducers of the EGFR pathway identifies BRAF-specific modulations of p-Erk1-2. (**a**) An example of the WB analysis of four samples; −control of basal activation (48 h old medium), + positive control (new culture medium incubated for 2 h), SB (new culture medium incubated for 2 h with 10 µM SB202190). Green rectangles highlight p-Erk1-2 modulation in BRAF wild-type organoids; the red rectangle indicates p-Erk1-2 in a BRAF-mutated organoid (OMCR18-025TK BRAF V600E). Orange rectangles highlight the inhibition of Akt phosphorylation. (**b**) Analysis of the fold increase in phosphorylation of EGFR, Akt and Erk1-2 in BRAF wild-type (wt) or -mutant (mut) organoids. Single cohorts (dots) and merged results (boxes) are reported. The green arrow indicates the powerful upregulation of p-Erk1-2 signaling induced by SB202190 in BRAF wt organoids as compared to its downregulation in BRAF-mutated ones (red arrow). The orange arrows indicate the inhibition of Akt phosphorylation by SB202190 in all samples. (**c**) Same conditions reported in b, comparing NRAS or KRAS (N-KRAS) wild-type and mutated organoids. Statistics: * *p* ≤ 0.05; *** *p* ≤ 0.001; n = 25.

The first cohort (eight organoid cultures; Figure 2a reports four examples) showed increased EGFR phosphorylation upon SB202190 treatment in all samples (2.308 ± 1.090), while Akt phosphorylation was always decreased (0.360 ± 0.284). Erk1-2 phosphorylation was increased in six out of eight samples (4.271 ± 2.165) and decreased in two out of eight (0.55 ± 0.004). Interestingly, p-Erk1-2 inhibition was not dependent on the p-EGFR status. While the increased phosphorylation of EGFR by SB202190 is in line with previous reports, the inhibitory activity exerted on p-Akt is apparently a new finding, though a possible influence on this pathway was proposed by Menon et al. [21]. The finding of two organoid cultures showing reduced Erk1-2 phosphorylation upon SB202190 treatment was not expected, as this molecule is specifically added to the organoid culture medium as an agonist of this pathway.

When the samples were analyzed according to their BRAF or N-KRAS mutation status (Figure 2b,c), it was immediately evident that the two cultures showing p-Erk1-2 inhibition upon SB202190 treatment were affected by BRAF mutations (Table 1; extended NGS data in Table S2). While one mutation was a canonical V600E substitution, the other one corresponded to a never-described duplication of V471, an amino acid previously reported as a rare target of active point mutations in metastatic CRC [22]. KRAS-NRAS mutations were not apparently involved in the EGFR, Akt or Erk1-2 phosphorylation status (Figure 2c).

The analysis of the second cohort (17 organoid cultures, Figure 2b,c) detected p-EGFR activation by SB202190 in most cultures (1.501 ± 0.655). Akt phosphorylation was inhibited in all samples (0.330 ± 0.220). Increased p-Erk levels were observed in 14/17 samples (4.086 ± 1.906) and decreased in 3 (0.465 ± 0.308). Even in this analysis, reduced p-Erk1-2 phosphorylation corresponded to the BRAF-mutated organoid cultures, with different activating hot-spots (V600E, K601E and G469V), while KRAS-NRAS mutations did not modify the EGFR, Akt or Erk1-2 phosphorylation status.

Overall, five out of five BRAF-mutated organoid cultures shared a striking downregulation of Erk1-2 signaling upon SB202190 treatment, despite the co-occurrence of increased p-EGFR signaling (Figure 2b). By contrast, the BRAF wild-type organoid cultures always showed strongly increased Erk1-2 signaling upon SB202190 treatment.

The NGS mutation analysis (Table 1 and Table S2) also demonstrated that our culture conditions did not select a particular mutational status in vitro. Indeed, two organoid cultures showed no detectable hot-spot mutation among the 22 genes of the NGS panel. The most frequent mutated target gene was TP53 (11/25 oncogenic and 4/25 non-validated variants), followed by KRAS (12/25 oncogenic and 1/25 inactivating variants). In addition, the analysis identified 10 novel gene variants: 4 missense mutations (1 CTNNB1, 1 ERBB2 and 2 ALK); 3 InDels (2 TP53 and 1 PTEN); and 3 variants in non-coding regions, such as splice sites (FBXW7 and PTEN) and 3′-UTR (PIK3CA). While five PIK3CA-mutated organoid cultures showed a modest or null increase in Akt signaling as compared to the other cultures, one organoid carrying double heterozygous truncating mutations of PTEN (T232N fsTer11; N323M fsTer21) showed huge basal p-Akt phosphorylation (not shown).

### 3.3. SB202190 Mimics the Activity of the BRAF V600E Inhibitor Dabrafenib on Erk1-2 Phosphorylation Status

The biochemical signature of SB202190 on p-Erk1-2 in BRAF wt/-mutated organoids resembled that of first-generation BRAF V600E inhibitors, known to inhibit Erk phosphorylation in BRAF-mutated tumors, but showing a paradoxical increase in p-Erk1-2 signaling in BRAF wild-type tumors [23,24]. We found that Lavoie et al. previously described the ability of the structurally similar p38 inhibitors SB202190 and SB203580 to trigger RAF dimerization [25]. To verify this drug mimicry, we tested in parallel our five BRAF-mutated organoids and seven non-mutated controls, comparing the effects of SB202190 with Dabrafenib mesylate (a first-generation BRAF V600E inhibitor, causing p-Erk1-2 activation in BRAF wild-type cells) and PLX8394 (a next-generation inhibitor, not inducing p-Erk1-2 activation) [26]. These conditions were tested in the presence or absence of EGF to measure the influence of the wild-type EGFR pathway on the overall effects. Moreover, the most specific p38 inhibitor available, BIRB796 [27], was tested to evaluate the selective involvement of p38 in p-Erk1-2 modulation.

**Figure 3:**
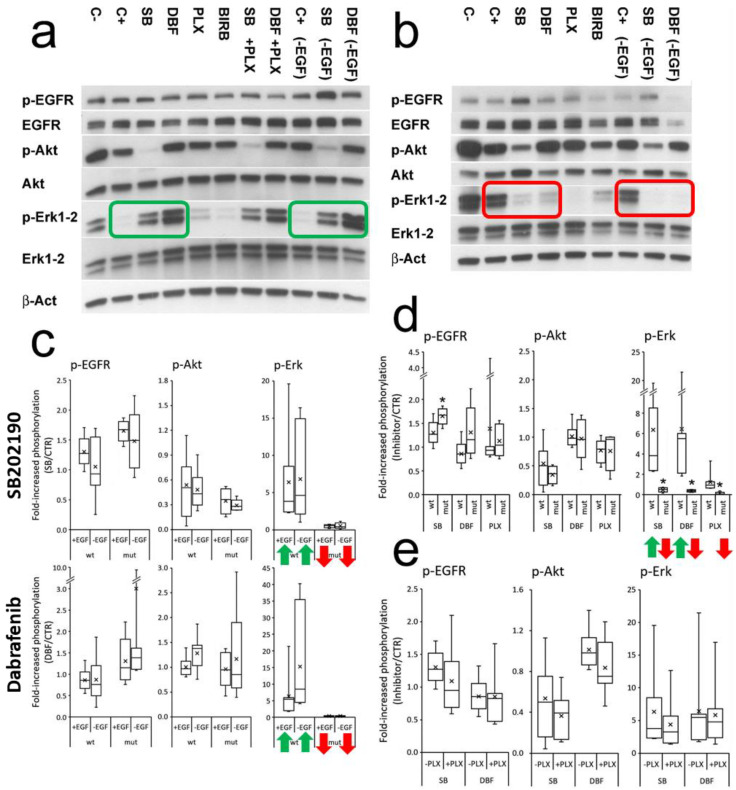
SB202190 mimics the biochemical activity of the BRAF inhibitor Dabrafenib on p-Erk1-2 modulation, unaffected by EGFR activation. (**a**) An example of the signaling of a BRAF wild-type organoid (OMCR18-035TK). The green rectangles highlight increased p-Erk1-2 activity induced by SB202190 and Dabrafenib, both in the presence (**left**) or absence (**right**) of EGF. (**b**) An example of the signaling of a BRAF mutated organoid (OMCR16-005TK, BRAF V600E). The red rectangles highlight the inhibition of p-Erk1-2 activity mediated by SB202190 and Dabrafenib, both in the presence (**left**) or absence (**right**) of EGF. Organoids were treated for 2 h in new culture medium with (C+) or without EGF (-EGF); 48 h old medium was used as control of basal activation (C−). SB202190 (SB) 10 µM, Dabrafenib (DBF) 1 µM, PLX8394 (PLX) 1 µM, BIRB796 (BIRB) 0.2 µM. (**c**) The effect of EGF (25 ng/mL) on SB202190- or Dabrafenib-treated organoids is represented for p-EGFR, p-Akt and p-Erk1-2, according to BRAF wild-type or -mutant status. No condition achieved statistically significant difference. The green arrows indicate the powerful upregulation of p-Erk1-2 signaling induced by SB202190 and Dabrafenib in BRAF wt organoids as compared to its downregulation in the BRAF-mutated ones (red arrows). (**d**) Comparison of the effects of SB, DBF and PLX on p-EGFR, p-Akt and p-Erk1-2, in BRAF wild-type or -mutant organoids. (**e**) Effects of PLX+SB or PLX+DBF co-treatment in BRAF wild-type organoids. Statistics: * *p* ≤ 0.05 (wt versus mutant BRAF), n = 12.

SB202190 and Dabrafenib always showed a coherent modulation of Erk1-2 phosphorylation according to the BRAF status, while PLX8394 acted as a powerful inhibitor of p-Erk1-2 in the BRAF-mutated organoids, without agonist effects in the non-mutated controls (Figure 3a,b,d). The presence/absence of EGF did not determine statistically significant changes in p-EGFR, p-Akt and p-Erk1-2 modulation by SB202190 or Dabrafenib (Figure 3c). The addition of PLX8394 along with SB202190 or Dabrafenib to the BRAF wt organoids did not revert p-Erk activation (Figure 3e). BIRB796 did not mimic SB202190 modulation, showing weak, variable activity on p-EGFR, p-Akt and p-Erk1-2, which was unrelated to the BRAF status and statistically not significant (Figure S2). These data indicate that SB202190 can modulate the EGFR pathway at different levels. While p-Erk1-2 can be modulated by BRAF-inhibitor mimicry, the effects on p-EGFR and p-Akt appear to be molecule-specific and not related to p38 inhibition.

### 3.4. Effects of SB202190, Dabrafenib and PLX8394 on CRC Organoid Growth

According to the inhibition of Erk phosphorylation in the BRAF-mutated organoids, SB202190, Dabrafenib and PLX8394 could exert a cytostatic/cytotoxic effect on these cultures. Moreover, Fujii and Sato [11] previously reported that some CRC organoid cultures were inhibited by the presence of SB202190. However, in patients with CRC, BRAF inhibitor monotherapy is inactive against the tumor [28]; thus, some efficacy would be unexpected.

We live-monitored the growth of our five BRAF-mutated organoid cultures and three controls, with a wild-type EGFR pathway, for nine days. We also quantified the ratio of dead/total cells at the end of the test to evaluate drug cytotoxicity.

**Figure 4:**
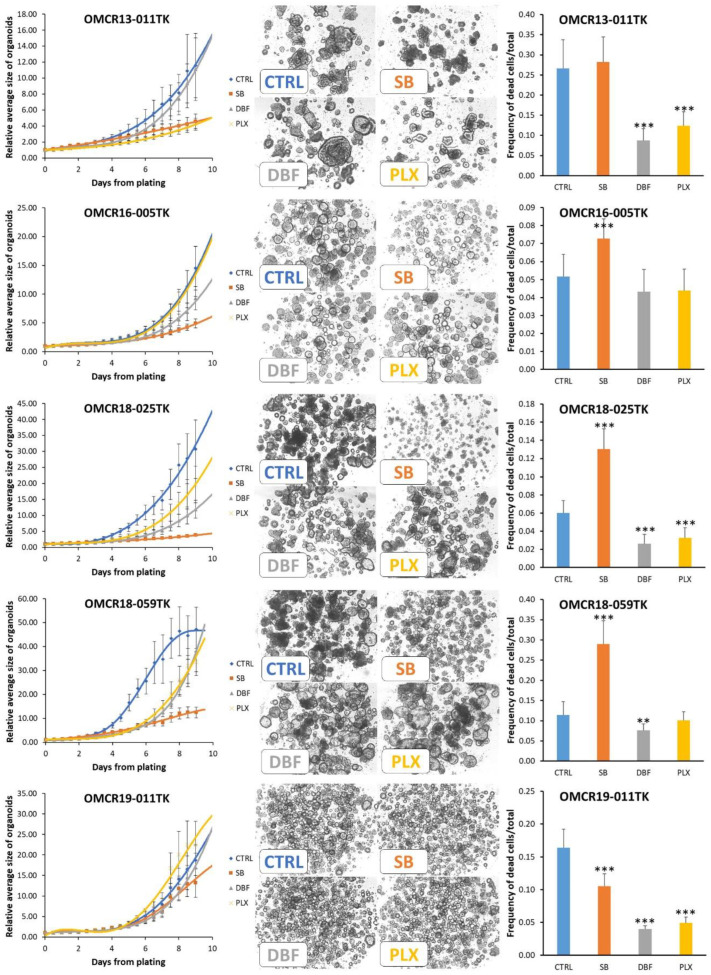
Modulation of BRAF-mutated organoid growth by SB202190 (10 µM), Dabrafenib (1 µM) and PLX8394 (1 µM). Left column: interpolation of the growth curves live-monitored for 9 days (12 h steps); all data are normalized against point 0 values to reduce variations in plating efficiency among different organoids. Each experimental point represents the mean of 4–5 replicates (geltrex domes) in three tests. Mutations: OMCR13-011TK BRAF K601E; OMCR16-005TK and OMCR18-025TK BRAF V600E; OMCR18-059TK BRAF Val471dup; OMCR19-011TK BRAF G469V. The statistics of each experimental point vs. controls (CTRL) are detailed in TabS3. Center column: example images from the last experimental point; the organoids monitored in a single geltrex dome are shown. Right column: the cytotoxic effects of drugs were assessed using live fluorescent marking and normalized against total cells (in the same tests shown in the left column). Statistics vs. controls (CTRL): ** *p* ≤ 0.01; *** *p* ≤ 0.001.

In the BRAF-mutated organoids, each organoid line showed a unique pattern of sensitivity to the tested drugs (Figure 4 and Table S3 for detailed statistics; Figure S3 shows a close-up of the first four days). However, in all cases, SB202190 showed the most stable inhibitory profile, with a reduced curve slope at the end of the test. This pattern of inhibition could indicate the inability of the organoids to biochemically contrast their activity due to the multiple targets of the drug (Erk, Akt and p38). Dabrafenib and PLX8394 activity was not superimposable. This finding is not unexpected, according to the different epitopes targeted in BRAF-mutated proteins (ATP pocket vs. dimerization domain) and the different patterns of additional mutations harbored by organoids. Dabrafenib was more efficacious in inhibiting OMCR16-005TK, OMCR18-025TK (BRAF V600E) and OMCR18-059TK (BRAF Val471dup) organoids. PLX8394 was more active on OMCR13-011TK (BRAF K601E), OMCR18-025TK and OMCR18-059TK organoids. OMCR19-011TK (BRAF G469V) showed a high resistance to therapy. SB202190 induced a significant increase in cytotoxicity in OMCR16-005TK, OMCR18-025TK and OMCR18-059TK organoids, while the opposite effect was exerted by Dabrafenib and PLX8394 on OMCR13-011TK, OMCR18-025TK and OMCR19-011TK, showing reduced cell death as compared to the controls. Thus, while SB202190 can contemporary exert an inhibitory and cytotoxic effect, Dabrafenib and PLX8394 may act as inhibitors of cell growth, and this metabolic slow-down could explain the rapid recovery after adaptation to therapy.

**Figure 5:**
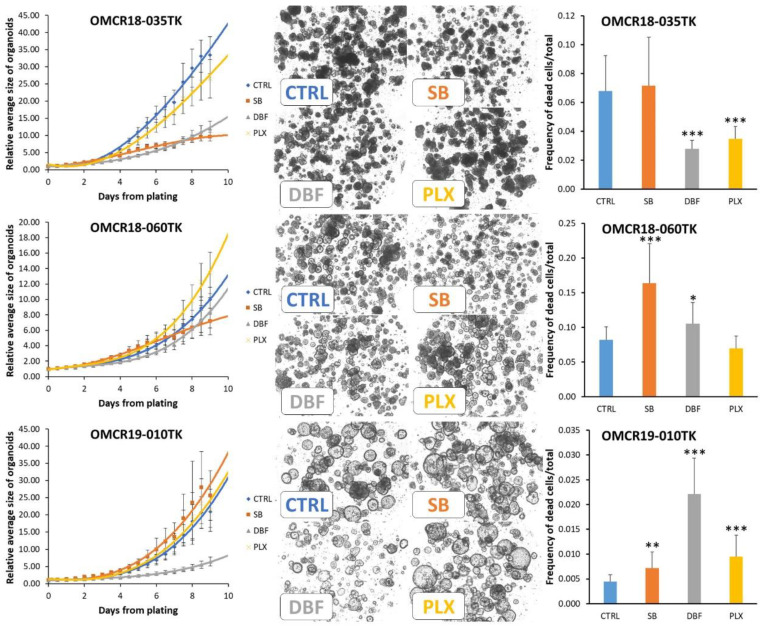
Organoids with wild-type EGFR signaling; modulation of growth by SB202190 (10 µM), Dabrafenib (1 µM) and PLX8394 (1 µM). Left column: interpolation of the growth curves live-monitored for 9 days (12 h steps); all data are normalized against point 0 values to reduce variations in plating efficiency among different organoids. Each experimental point represents the mean of 4–5 replicates (geltrex domes) in three tests. Mutations: OMCR18-035TK (frame-shift mutation of TP53 with undefined effect); OMCR18-060TK (no mutation in our NGS panel); and OMCR19-010TK (double PTEN truncation, undescribed ALK mutation, TP53 R181H). The statistics of each experimental point vs. controls (CTRL) are detailed in TabS3. Center column: example images from the last experimental point; the organoids monitored in a single geltrex dome are shown. Right column: the cytotoxic effects of drugs were assessed using live fluorescent marking and normalized against total cells (in the same tests shown in the left column). Statistics vs. controls (CTRL): * *p* ≤ 0.05; ** *p* ≤ 0.01; *** *p* ≤ 0.001.

The analysis of the three organoids with wild-type EGFR signaling showed some unexpected results, as both SB202190 and Dabrafenib exerted biologically relevant, inhibitory effects (Figure 5). OMCR18-035TK and OMCR18-060TK were inhibited by SB202190, with the latter organoid showing a retarded response to the drug. Notably, the organoids with less/no response to SB202190, namely, OMCR19-011TK (BRAF mutated) and OMCR19-010TK, carried a PIK3CA activating mutation (M1043V) and truncated PTEN (T232N fsTer11; N323M fsTer21), respectively. As both these hits are involved in Akt signaling, this might suggest a primary role of Akt inhibition in SB202190 activity, a hypothesis deserving further investigations.

OMCR18-035TK and OMCR19-010TK were sensitive to Dabrafenib. This inhibitory effect is difficult to explain. However, Phadke et al. previously observed similar activity in BRAF wild-type melanoma cell cultures [26]. In that model, Dabrafenib, but not Vemurafenib, specifically targeted NEK9 and CDK16, causing G0-G1 arrest. Finally, PLX8394 caused a reduced alteration in organoid growth. The evaluation of drug cytotoxicity showed variable results, with SB202190 doubling cell death in OMCR18-060TK. The cytotoxic effects on OMCR19-010TK organoids are of scarce biologic relevance, despite them having a strong statistical significance, according to the low number of cells affected.

## 4. Discussion

Primary colorectal cancer organoids are fundamental tools for in vitro studies and useful alternatives to in vivo tests. CRC organoids are able to self-organize into three-dimensional crypt-like structures of pure epithelial cells with different phenotypes, and they are sensitive to environmental stimuli able to promote their stemness and differentiation [11,29]. While CRC organoids may be the gold standard for any in vitro study involving this cancer, their diffusion as a basic tool is still limited, and the culture methods are not completely standardized. The goal of our study was to carry out biochemical characterization of the signaling induced by SB202190. Fujii et al. [11] tested different culture conditions on 28 CRC samples (giving rise to 37 distinct organoid cultures): a medium with or without SB202190 (10 µM), hypoxia and/or wnt3a + R-spondin. Among the primary cultures obtained, 14 cultures (37.8%) needed SB202190 for expansion, while 7 (18.9%) were inhibited. The authors linked SB202190’s promoting activity to EGFR signaling stabilization as a trigger of Erk1-2 phosphorylation [11]. Accordingly, they suggested that SB202190 could positively affect CRC organoid cultures without KRAS-activating mutations, relying on canonical EGFR signaling, while no explanation was hypothesized for SB202190’s inhibitory effect.

In our conditions, SB202190 was unnecessary for culturing CRC organoids (with only one exception), including cultures showing no mutation in the EGFR pathway, according to our NGS panel. An SB202190-induced p-Erk1-2 increase was always observed in the absence of BRAF mutations, even in Her2-, KRAS- and NRAS-mutated organoids, suggesting the presence of a downstream target for the inhibitor. In the presence of SB202190, the p-eErk1-2 status was not apparently related to EGFR activation or stabilization; in fact, it did not rely on exogenous EGF addition. Moreover, Erk1-2 signaling was switched off in the BRAF-mutated organoids, despite them having a significantly increased p-EGFR stabilization as compared to the BRAF wild-type cultures.

The intriguing biochemical signature of SB202190 allowed for the prediction of different BRAF-activating mutations in the CRC organoids before the NGS analysis. While this signature could have immediate diagnostic and possible therapeutic implications, the underlying mechanism of SB202190 activity was not clear. The findings by Lavoie et al. [25] allowed us to identify a possible explanation for our data: these authors previously described the ability of SB202190 to trigger BRAF/CRAF dimerization. RAF dimerization is also induced by first-generation BRAF V600E inhibitors in melanoma models, inducing the paradoxical activation of p-Erk1-2 in BRAF wild-type cultures [23]. Indeed, when we compared SB202190 to the BRAF inhibitor Dabrafenib, we found a perfect correspondence between p-Erk1-2 signaling and the BRAF mutational status.

In our experimental conditions, SB202190 increased organoid proliferation in only one culture. This was an unpredicted observation, considering the strong amplification of Erk1-2 phosphorylation observed in all the BRAF wild-type cultures. Indeed, the role of p-Erk1-2 signaling as a mediator of colorectal crypt homeostasis is quite complex; while its primary role may be proliferation induction [10], it also affects BMPs/Smad signaling by the inhibitory phosphorylation of S206 in the Smad1 linker region [30,31]. While the blocking of BMP signaling would positively affect crypt stemness, a recent study by Zahn et al. [32] reported that the inhibition of Erk1-2 phosphorylation by MEK inhibitors results in increased wnt activity and stemness induction in CRC organoids. Thus, p-Erk1-2 signaling amplification/inhibition could trigger opposing effects on the stem cell niche of CRC.

A unique feature of SB202190 was the ability to reduce Akt signaling in all the organoid cultures. This inhibition could be the basis of the enforced, long-term inhibition of organoid growth, as compared to BRAF inhibitors. While this is only a hypothesis, indeed, two organoid cultures almost unaffected by SB202190 treatment carried PIK3CA-activating or PTEN-truncating mutations, both involved in p-Akt induction.

Another interesting finding linked to our biochemical analysis is the proof of the biological activity of new, yet undescribed mutations. Indeed, BRAF Val471 duplication in OMCR18-059TK was immediately verified to be active by SB202190 treatment, while OMCR19-010TK PTEN mutations were immediately revealed to be active by huge p-Akt signaling. Thus, the biochemistry of signaling pathways in primary organoids could implement patient-specific theranostics with unique information.

## 5. Conclusions

The inhibition of p-Erk1-2 by SB202190 in a specific organoid can predict the activating mutation of BRAF before genomic analyses, and it can validate it for inclusion in COSMIC and other databases. This biochemical signature mimics the behavior of first-generation BRAF inhibitors and does not directly involve EGFR stabilization.

Since SB202190 reduces p-Akt signaling, this off-target effect, along with p38 and Erk1-2 inhibition in BRAF-mutated CRC organoids, could be the first step for the development of new multitarget drugs.

Overall, our study shows how the biochemistry of patient-derived organoids can implement genomic data with unique information about the biological activity of drugs and the validation of new driving mutations, two necessary conditions for personalized therapy.

## Figures and Tables

**Table 1:** Mutations and splice variants in organoids, detected by NGS, and basic pathology data of originating tumors. Oncogenic and truncating mutations of oncosuppressor genes with known activity are reported in red bold.

ID	Set	Loc	MS Status	Stage UICC	T	N	M	G	Molecular Diagnostic of Tumor Tissue	Mutation Status by NGS												
										CTNNB1	FBXW7	MET	ERBB2	KRAS	NRAS	BRAF	DDR2	PIK3CA	PTEN	ALK	SMAD4	TP53
**OMCR17-062TK**	Exp	T	MSS	III	3	2a		3							**Q61R**			*3236T>C*				**R248Q**
**OMCR17-068TK**	Exp	L	MSS	III	3	1b		2													*TACTT->A*	**R273C**
**OMCR18-016TK**	Exp	T	**MSI**	II	3	0		2			**R465H**		**L755S**									
**OMCR18-021TK**	Exp	S	MSS	IV	3	2a	1a	2	*KRAS* G12V					**G12V**								
**OMCR18-025TK**	Exp	R	MSS	I	2	0		2		**G34E**	**R505C**					**V600E**						
**OMCR18-035TK**	Exp	R	MSS	II	4b	0		2														S241 fs*22
**OMCR18-059TK**	Exp	RT	MSS	I	2	0		2						G60V		V471dup						N131 del
**OMCR18-060TK**	Exp	RT	MSS	II	3	0		2	*KRAS/BRAF/NRAS* WT													
**OMCR19-003TK**	Val	R	MSS	III	2	1b		3	*KRAS* G12A				N873G	**G12A**								**R282W**
**OMCR19-006TK**	Val	RT	MSS	I	2	0		2			*1418+1G>A*			**A59T**						R1212H		**R196P**
**OMCR19-009TK**	Val	R	MSS	III	4a	2a		3	*KRAS* G12R; *PIK3CA* Q546K					**G12R**				**Q546K**				**R273C**
**OMCR19-010TK**	Val	T	MSS	II	3	0		1											T232N fsTer11; N323M fsTer21	L1165H		**R181H**
**OMCR19-011TK**	Val	R	MSS	III	3	1b		3						**A146P**		**G469V**		**M1043V**				**R282W**
**OMCR19-015TK**	Val	S	MSS	III	3	1b		2	*KRAS* G12A			R988C		**G12A**								
**OMCR19-016TK**	Val	R	**MSI**	II	3	0		3	*BRAF wt*	**T41A**								**E545K**	*801+2T>C*			P152R fsTer18
**OMCR19-017TK**	Val	RT	MSS	II	3	0		2														
**OMCR19-024TK**	Val	T	MSS	II	3	0		2						**G12C**			**M441I**					
**OMCR19-030TK**	Val	T	MSS	III	3	2a		2	*KRAS* G12D					**G12D**							**R135 Ter**	Q104R fsTer19
**OMCR19-034TK**	Val	R	MSS	IV	3	2b	1a	2	*KRAS* G13V					**G13V**				**E542K**				**R175H**
**OMCR19-040TK**	Val	R	MSS	III	3	2a		2						**G12A**								**R175H**
**OMCR19-041TK**	Val	R	MSS	III	3	2a		3			**R505C**			**G12V**				**G1049R**			**S178 Ter**	**R213 Ter**
**OMCR20-002TK**	Val	R	MSS	II	3	0		2						**G12D**								
**OMCR13-011TK**	Ad	R	MSS	III	4a	2b		3	*BRAF* K601E							**K601E**						
**OMCR15-045TK**	Ad	R	MSS	II	3	0		2				**N375S**		**G12V**								**R282W**
**OMCR16-005TK**	Ad	R	MSS	IV	4a	2a	1b	4	*BRAF* V600E	S23N						**V600E**						**G244S**

Table legend: Exp, experimental set; Val, validation set; Ad, added cases. Loc, localization of primary tumor; R, right colon; T, transverse colon; L, left colon; S, sigma; RT, rectum. MS, microsatellite; MSS, microsatellite-stable tumor; MSI, microsatellite-unstable tumor.

## Data Availability

The datasets generated and analyzed during the current study are available in the Sequence Read Archive, BioProject ID: PRJNA759362, https://www.ncbi.nlm.nih.gov/sra.

## Supplementary Materials

The following supporting information can be downloaded at https://www.mdpi.com/article/10.3390/cells12040664/s1, Figure S1: Sanger validation of BRAF mutations (PDF); Figure S2: The specific P38 inhibitor BIRB796 does not mimic SB202190 activity; Figure S3: A close-up of organoid growth during the first 4 days of the test. Table S1: Patients’ cohort and establishment of organoid cultures (XLS); Table S2: Detailed NGS analysis of organoid cultures (XLS); Table S3: Statistics of data reported in Figure 4 and Figure 5 (PDF).
