# Supplementary material for: SB202190 Predicts BRAF-Activating Mutations in Primary Colorectal Cancer Organoids via Erk1-2 Modulation
Source: Cells. 2023 Feb 20;12(4):664. doi: 10.3390/cells12040664 (PMC9954675; doi:10.3390/cells12040664)
Supplement: Supplementary file 1 [file cells-12-00664-s001.zip › Figure S1 (Sanger-Cells-corrected).pdf]

Figure S1: Sanger validation of BRAF mutations

Sample: OMCR18-059TK

NGS

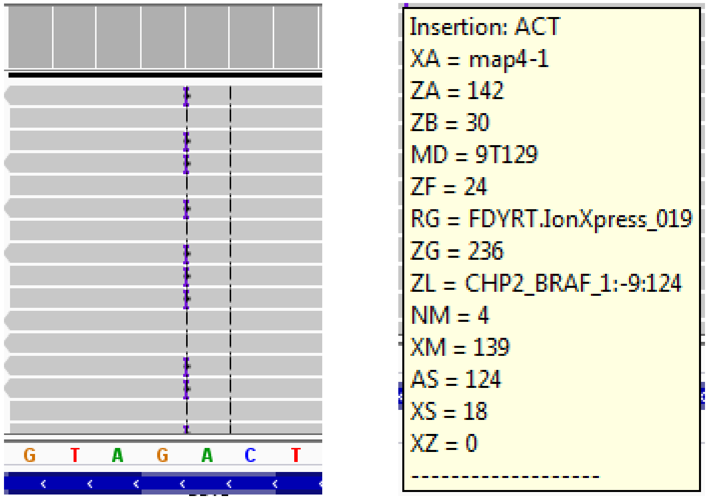

Sanger Seq

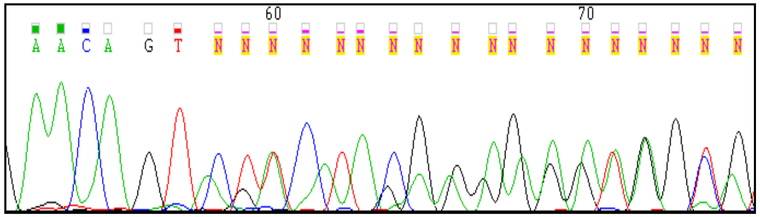

BRAF **c. 1412_1413insAGT; BRAF p.V471dup**

Sample: OMCR13-011TK

NGS

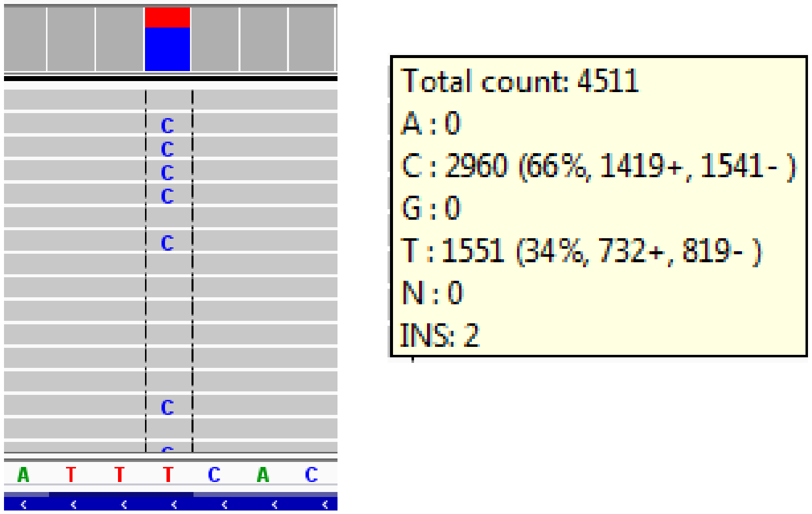

Sanger Seq

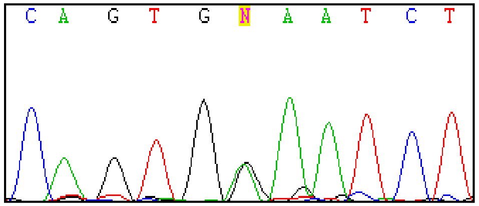

BRAF **c.1801A>G;** ***BRAF*** **p.K601E**

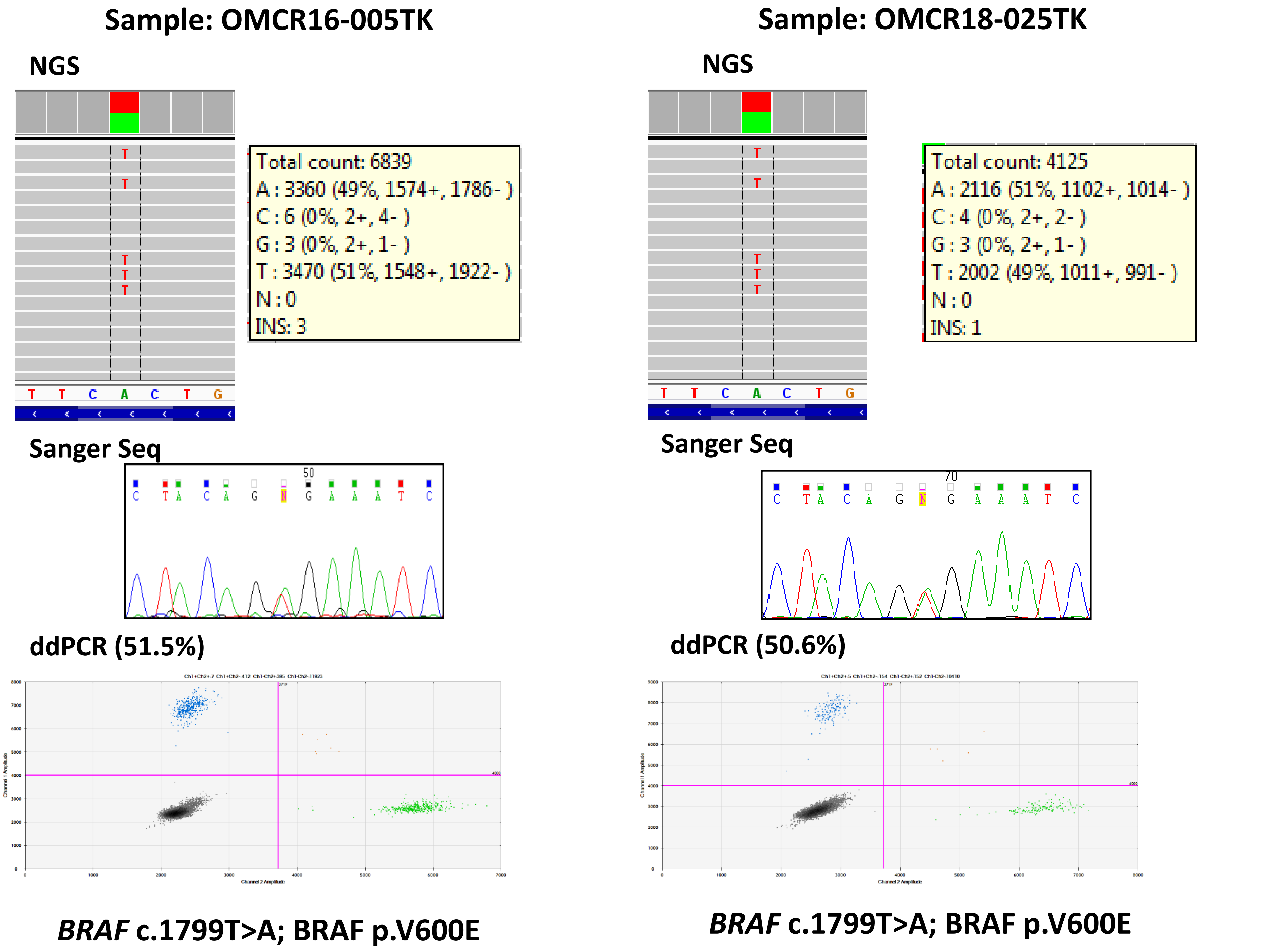
Sample: OMCR16-005TK
NGS
Total count: 6839
A : 3360 (49%, 1574+, 1786-)
C : 6 (0%, 2+, 4-)
G : 3 (0%, 2+, 1-)
T : 3470 (51%, 1548+, 1922-)
N : 0
INS: 3
Sanger Seq
ddPCR (51.5%)
BRAF c.1799T>A; BRAF p.V600E
Sample: OMCR18-025TK
NGS
Total count: 4125
A : 2116 (51%, 1102+, 1014-)
C : 4 (0%, 2+, 2-)
G : 3 (0%, 2+, 1-)
T : 2002 (49%, 1011+, 991-)
N : 0
INS: 1
Sanger Seq
ddPCR (50.6%)
BRAF c.1799T>A; BRAF p.V600E

Sample: OMCR19-011TK

NGS

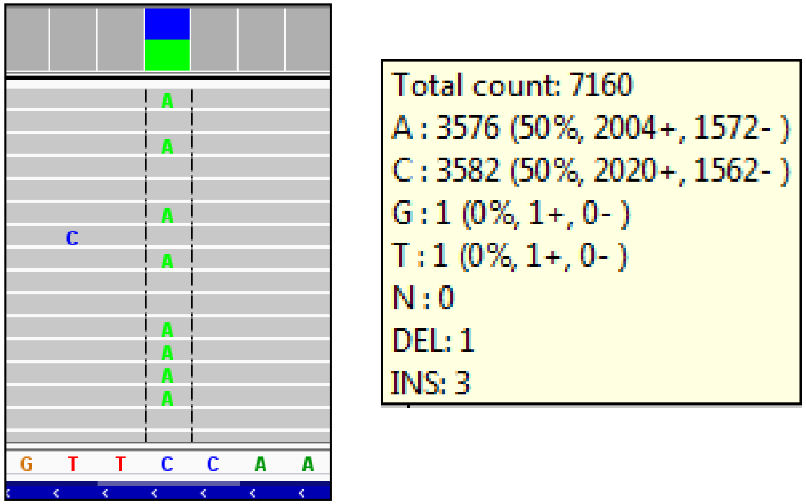

Sanger Seq

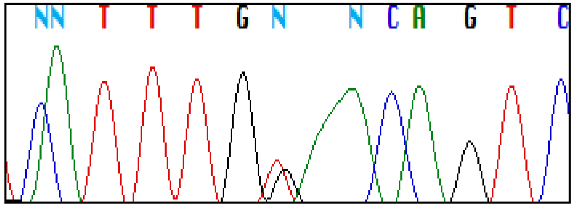

BRAF **c.1406G>T; BRAF p.G469V**

Sample: OMCR19-060TK

NGS

Wild Type

Sanger Seq

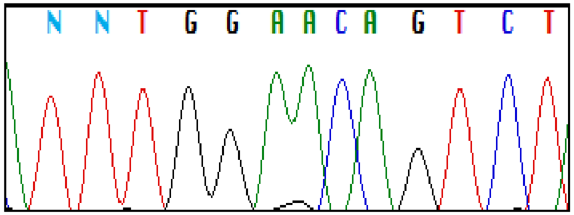

BRAF **Wild-type; BRAF Wild-type**
