# Supplementary material for: SB202190 Predicts BRAF-Activating Mutations in Primary Colorectal Cancer Organoids via Erk1-2 Modulation
Source: Cells. 2023 Feb 20;12(4):664. doi: 10.3390/cells12040664 (PMC9954675; doi:10.3390/cells12040664)
Supplement: Supplementary file 1 [file cells-12-00664-s001.zip › Table S3 (Statistics-Cells).pdf]

Table S3. T-Test statistics form data represented in fig.4 and 5 (organoids growth).

Red dot p>0.05; Yellow dot 0.01≤p≤0.05 *; Green dot p<0.01 ** or ***

	BRAF-mutated organoids					EGFR pathway wild-type organoids		
	**OMCR13-011TK**	**OMCR16-005TK**	**OMCR18-025TK**	**OMCR18-059TK**	**OMCR19-011TK**	**OMCR18-035TK**	**OMCR18-060TK**	**OMCR19-010TK**
**SB vs CTR**	*p=*	*p=*	*p=*	*p=*	*p=*	*p=*	*p=*	*p=*
0.5	Red 5.3235E-04	Red 8.7531E-01	Yellow 3.9418E-04	Red 2.6135E-01	Red 1.1984E-01	Red 6.2099E-02	Red 2.6623E-02	Green 9.3817E-05
1.0	Yellow 2.2139E-04	Red 1.8948E-01	Green 8.6383E-05	Red 1.9484E-01	Red 5.6388E-01	Red 7.7289E-02	Red 6.1185E-02	Green 1.2739E-07
1.5	Red 1.2976E-03	Red 2.5056E-03	Red 8.5685E-03	Red 5.5511E-01	Red 3.7155E-01	Red 3.7862E-01	Yellow 1.7017E-04	Green 1.4343E-06
2.0	Red 2.5929E-01	Red 5.5822E-04	Red 6.9611E-01	Red 8.5728E-02	Red 7.2542E-01	Red 4.5068E-03	Red 4.0765E-03	Green 2.7156E-06
2.5	Red 4.8304E-01	Green 4.8077E-05	Red 8.4610E-02	Red 4.8636E-03	Red 7.0078E-01	Red 3.7790E-02	Green 4.8724E-05	Green 1.6083E-06
3.0	Red 2.4971E-01	Green 1.8543E-06	Green 3.2373E-05	Red 5.2493E-04	Red 9.3575E-01	Green 4.6054E-05	Green 6.3509E-05	Green 7.7925E-06
3.5	Red 1.4359E-01	Green 5.1917E-06	Green 2.4538E-07	Green 9.2890E-06	Red 9.0887E-01	Green 1.7894E-06	Green 4.8786E-05	Green 7.1549E-07
4.0	Red 7.6335E-02	Green 5.4902E-06	Green 1.0182E-07	Green 2.0807E-07	Red 9.2935E-01	Green 4.7321E-12	Red 4.3581E-03	Red 1.3423E-03
4.5	Red 2.2895E-02	Green 1.5149E-06	Green 2.2923E-08	Green 1.0746E-09	Red 5.0255E-01	Green 3.4536E-12	Yellow 2.4376E-04	Yellow 2.3413E-04
5.0	Red 3.6319E-03	Green 2.2644E-07	Green 1.5892E-08	Green 8.2336E-09	Red 2.8149E-02	Green 2.4312E-10	Yellow 1.9580E-04	Yellow 4.3953E-04
5.5	Red 1.2362E-03	Green 5.8902E-08	Green 1.0034E-09	Green 2.5111E-10	Red 5.0258E-02	Green 8.2891E-12	Red 1.3416E-03	Red 1.7352E-03
6.0	Yellow 2.1858E-04	Green 1.3440E-07	Green 3.1959E-09	Green 6.2119E-09	Red 5.0440E-02	Green 1.9466E-10	Red 3.0553E-02	Red 4.9056E-03
6.5	Yellow 2.1187E-04	Green 8.9563E-08	Green 4.1119E-09	Green 5.9869E-08	Red 3.0499E-02	Green 8.2564E-10	Red 1.5517E-01	Red 4.1497E-03
7.0	Green 8.6875E-05	Green 1.5540E-08	Green 1.6478E-09	Green 1.6779E-07	Red 8.2171E-04	Green 4.5077E-09	Red 1.6784E-01	Red 3.9379E-02
7.5	Green 8.1921E-06	Green 1.2704E-08	Green 6.2978E-09	Green 6.2731E-10	Red 2.6800E-02	Green 1.3968E-08	Red 3.2865E-02	Red 3.0071E-02
8.0	Green 1.0427E-05	Green 2.7223E-08	Green 1.0485E-08	Green 5.3159E-09	Red 4.4076E-02	Green 1.9033E-09	Red 3.4285E-02	Red 1.3418E-01
8.5	Yellow 1.1686E-04	Green 2.4831E-07	Green 2.7801E-08	Green 4.6819E-10	Red 7.7665E-03	Green 1.6812E-11	Red 3.8304E-03	Red 2.9550E-02
9.0	Green 1.7510E-05	Green 2.0927E-07	Green 5.3597E-08	Green 1.1542E-09	Yellow 4.4019E-04	Green 1.3910E-10	Yellow 1.5216E-04	Red 5.0980E-02
**DBF vs CTR**	*p=*	*p=*	*p=*	*p=*	*p=*	*p=*	*p=*	*p=*
0.5	Red 2.1429E-01	Red 2.5188E-01	Red 7.6765E-01	Red 2.8953E-02	Red 2.4777E-01	Red 3.2037E-01	Red 3.8831E-01	Red 3.5082E-01
1.0	Red 1.5990E-02	Red 1.1279E-01	Red 8.8631E-02	Green 1.4181E-06	Red 8.4776E-01	Green 2.5996E-09	Red 1.8685E-01	Red 2.8077E-03
1.5	Yellow 2.0084E-04	Red 1.2091E-02	Red 1.2522E-02	Green 2.1323E-09	Red 8.4132E-01	Green 5.5596E-11	Red 2.3018E-01	Yellow 2.0032E-04
2.0	Yellow 1.0151E-04	Red 8.4271E-03	Yellow 1.3300E-04	Green 4.9182E-09	Red 8.2288E-01	Green 6.7653E-15	Red 3.0812E-02	Green 1.3767E-07
2.5	Green 1.9628E-05	Red 9.7950E-04	Green 8.2598E-06	Green 2.1577E-09	Red 2.4618E-01	Green 2.8148E-14	Red 3.0102E-02	Green 1.2905E-08
3.0	Green 5.6049E-06	Green 3.4964E-05	Green 1.5954E-06	Green 1.9117E-08	Red 9.9810E-02	Green 1.1022E-15	Red 1.1119E-02	Green 2.0060E-11
3.5	Green 6.5107E-05	Green 4.5701E-05	Green 1.2490E-07	Green 3.3765E-09	Red 2.4628E-02	Green 7.6319E-17	Red 2.3425E-03	Green 5.9356E-10
4.0	Yellow 1.1547E-04	Green 3.3750E-05	Green 1.4560E-07	Green 8.0809E-09	Red 3.8316E-02	Green 2.6434E-16	Green 6.2423E-06	Green 2.4524E-10

4.5	2.3551E-04	2.0343E-05	2.5904E-08	3.2200E-10	4.4991E-02	8.3889E-14	1.9290E-05	6.8484E-09
5.0	1.2429E-03	2.4893E-06	3.6605E-08	6.9522E-09	8.3000E-04	2.0012E-11	1.7584E-05	1.3966E-08
5.5	2.7322E-03	1.4796E-06	1.7806E-09	4.4789E-10	4.4532E-03	2.0074E-12	9.6813E-05	6.7678E-09
6.0	4.5353E-03	4.0256E-06	7.1821E-09	9.5269E-09	2.7795E-03	5.9885E-11	3.3752E-05	3.6385E-08
6.5	1.5750E-02	9.1679E-06	1.2552E-08	1.5934E-07	8.3471E-03	3.5275E-10	5.7711E-05	5.2563E-09
7.0	6.0915E-02	4.9227E-06	5.2600E-09	1.2292E-06	7.9186E-04	3.1053E-09	9.2023E-06	1.2869E-09
7.5	1.7158E-01	2.5484E-06	4.4423E-08	7.1459E-09	5.8079E-04	1.3275E-08	6.6552E-04	4.5161E-09
8.0	3.6041E-01	5.4844E-06	9.9027E-08	3.8799E-07	1.7437E-03	1.7892E-09	1.1575E-02	1.2258E-07
8.5	2.7876E-01	4.2597E-05	4.7217E-07	6.4452E-06	1.0397E-01	1.3574E-11	3.4403E-02	6.4860E-09
9.0	7.5664E-01	1.9144E-04	1.9303E-06	1.5966E-02	1.8910E-01	1.1213E-10	1.9274E-02	6.8200E-09
**PLX vs CTR**	*p=*	*p=*	*p=*	*p=*	*p=*	*p=*	*p=*	*p=*
0.5	4.6997E-02	8.8651E-01	8.2607E-01	2.6355E-02	3.7420E-01	8.0641E-01	2.2575E-01	8.3041E-01
1.0	1.7216E-05	6.3289E-01	7.2143E-01	9.9401E-06	4.7364E-01	1.4079E-05	3.8032E-01	5.4617E-01
1.5	5.6436E-07	2.6671E-01	1.3059E-01	4.0420E-09	5.0451E-01	4.2226E-05	6.9132E-02	8.2845E-01
2.0	8.4713E-07	2.6669E-01	4.9477E-03	3.8461E-09	2.3450E-01	9.3381E-11	4.3140E-01	8.9256E-01
2.5	1.4070E-07	2.3688E-01	1.0270E-03	2.4925E-10	3.8999E-01	1.2049E-06	4.2131E-02	9.7874E-01
3.0	2.3556E-08	2.2703E-02	6.3933E-05	6.8176E-09	1.9991E-01	4.6006E-07	7.2965E-02	8.6123E-01
3.5	2.4319E-07	7.4527E-02	5.6487E-06	8.8645E-10	1.1552E-01	4.4954E-07	8.5224E-03	2.5233E-01
4.0	1.7071E-07	9.5575E-02	2.4732E-06	3.4836E-09	4.3781E-02	2.2613E-08	3.3538E-02	8.6796E-01
4.5	3.9173E-07	1.4537E-01	5.6175E-07	1.5175E-10	3.3866E-02	4.4458E-08	1.8086E-03	5.3522E-01
5.0	4.1436E-06	1.8135E-01	6.0931E-07	4.6464E-09	1.0102E-01	7.5879E-06	3.5666E-04	4.6231E-01
5.5	6.1911E-06	4.2559E-01	2.1495E-08	2.1511E-10	5.2727E-03	5.0211E-06	3.6799E-04	5.5154E-01
6.0	3.8375E-06	4.1167E-01	1.0886E-07	4.4600E-09	4.3875E-03	2.5694E-04	4.2706E-04	5.7778E-01
6.5	8.5203E-06	4.7054E-01	7.8041E-07	1.5713E-07	2.7783E-03	2.2365E-03	2.0217E-04	1.5405E-01
7.0	1.5407E-05	2.8423E-01	1.7367E-06	1.9780E-06	4.5681E-02	2.7979E-02	7.5686E-03	4.5669E-01
7.5	1.7906E-06	3.4703E-01	5.1746E-06	1.1969E-08	4.9168E-04	1.6108E-02	1.3546E-03	2.3067E-01
8.0	2.7575E-06	7.0422E-01	8.8508E-06	1.9853E-06	2.0288E-03	1.2521E-03	9.6068E-04	5.1465E-01
8.5	6.2796E-05	7.0799E-01	1.5062E-04	2.1193E-04	3.4976E-03	5.5230E-04	1.5326E-04	2.7640E-01
9.0	1.0696E-05	7.2418E-01	8.6538E-04	1.1242E-04	5.7078E-02	2.8631E-03	4.1878E-04	8.4921E-01
