# Supplementary figures and images for: SB202190 Predicts BRAF-Activating Mutations in Primary Colorectal Cancer Organoids via Erk1-2 Modulation
Source: Cells. 2023 Feb 20;12(4):664. doi: 10.3390/cells12040664 (PMC9954675; doi:10.3390/cells12040664)

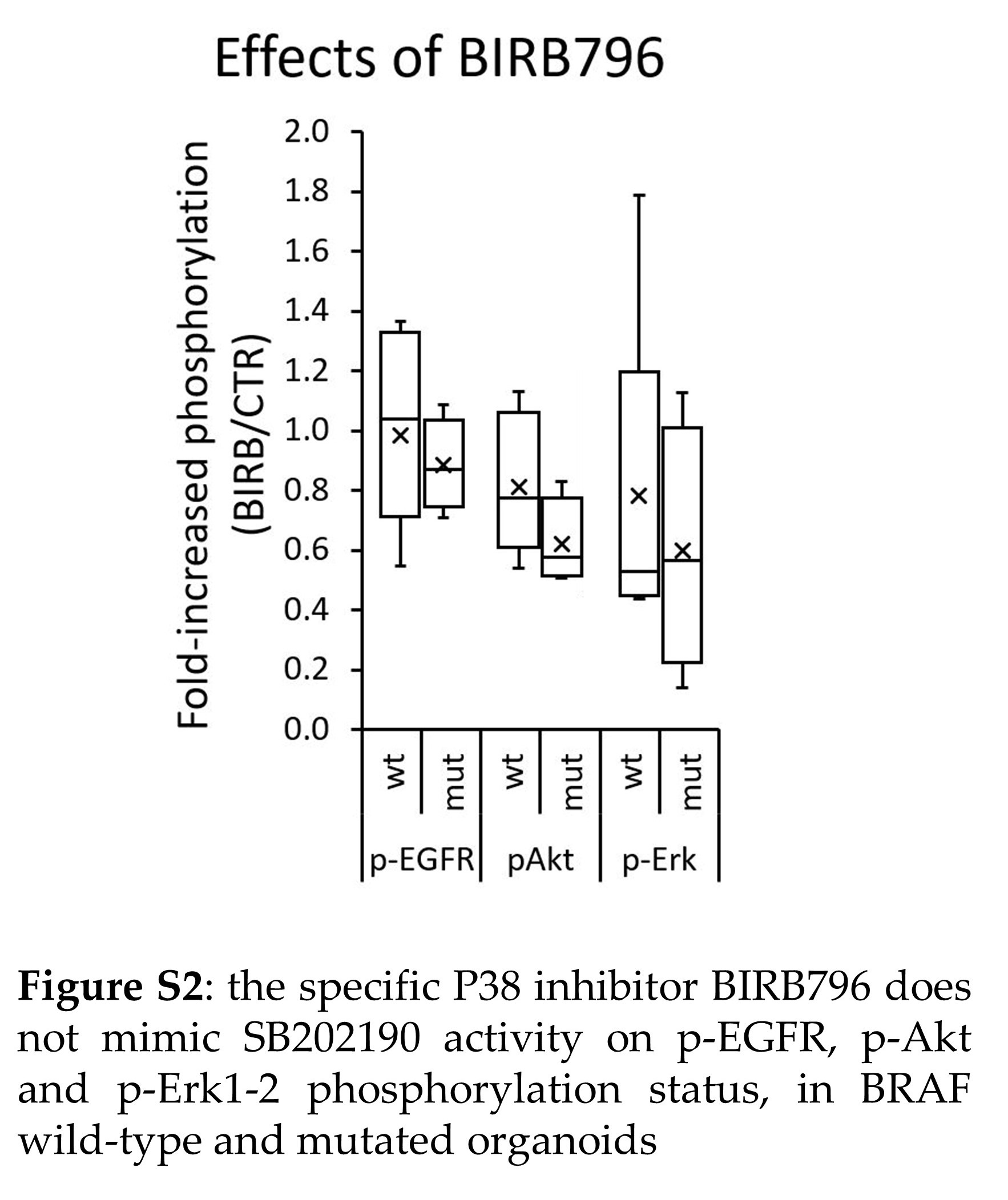

Supplement: Supplementary file 1 [file cells-12-00664-s001.zip › Figure S2 (Cells).jpg]

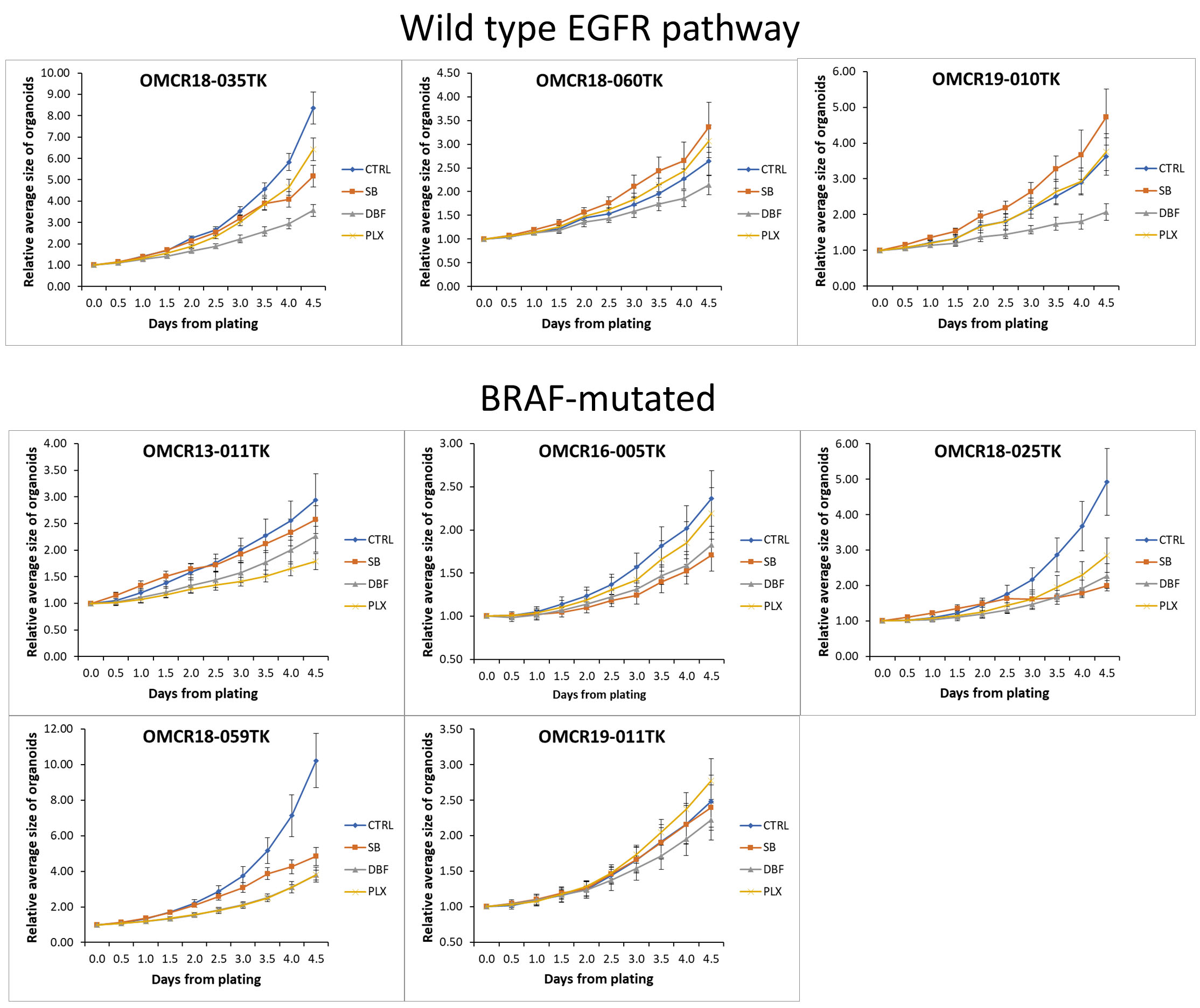

Supplement: Supplementary file 1 [file cells-12-00664-s001.zip › Figure S3.jpg]
